# The patient experience of sleep problems and their treatment in the context of current delusions and hallucinations

**DOI:** 10.1111/papt.12073

**Published:** 2015-08-18

**Authors:** Felicity Waite, Nicole Evans, Elissa Myers, Helen Startup, Rachel Lister, Allison G. Harvey, Daniel Freeman

**Affiliations:** ^1^ Department of PsychiatryWarneford HospitalUniversity of OxfordUK; ^2^ East London NHS Foundation TrustLondonUK; ^3^ Sussex Partnership NHS Foundation TrustSussexUK; ^4^ University of OxfordUK; ^5^ Department of PsychologyUniversity of CaliforniaBerkeleyCaliforniaUSA

**Keywords:** schizophrenia, insomnia, qualitative, psychological therapy

## Abstract

**Objectives:**

There is increasing recognition that sleep problems are common in patients with psychosis, that they exacerbate delusions and hallucinations and should be a treatment target. The aim of this study was to gain a patient perspective on the nature of sleep problems in psychosis and experience of treatment.

**Design:**

A qualitative, semi‐structured interview‐based study to explore patient accounts of sleep problems and associated psychological treatment.

**Methods:**

Ten patients with recent delusions and hallucinations, who had experienced sleep problems and received psychological treatment during a clinical trial (the Better Sleep Trial), were interviewed. Responses were analysed using interpretative phenomenological analysis.

**Results:**

Patients reported experiencing problems of getting to sleep, staying asleep, too much sleep, nightmares, and erratic sleep patterns. These sleep problems caused emotional distress, fatigue, and reduction in daytime activities. Worry and psychotic experiences disturbed sleep, while consequent tiredness meant that patients coped poorly with voices and persecutory fears. Treatment for sleep problems was viewed very positively and considered to have wide‐ranging impacts.

**Conclusions:**

Sleep disturbance is a major problem for patients with psychosis, which should be treated more often in services using evidence‐based interventions.

**Practitioner points:**

- Psychological interventions for sleep problems are valued by patients with psychosis.
- Patients with current distressing psychotic experiences report wide‐ranging benefits from a brief psychological intervention for sleep problems.

## Background

Sleep disturbance may be a contributory cause, rather than a consequence, of many mental health problems (Anderson & Bradley, 2013; Baglioni *et al*., 2011; Harvey, 2008). In patients with psychosis the presence of sleep problems, including difficulties getting and staying asleep (Monti & Monti, 2005) and circadian rhythm disruption (Wulff, Dijk, Middleton, Foster, & Joyce, 2012), have been documented. Indeed, over half of patients with persecutory delusions report insomnia (Freeman, Pugh, Vorontsova, & Southgate, 2009). Problems with sleep predict the occurrence of psychotic experiences, low quality of life, and impaired coping (Freeman *et al*., 2012; Hofstetter, Lysaker, & Mayeda, 2005; Ruhrmann *et al*., 2010). Moreover, treating the sleep problems experienced by patients with psychosis is important in its own right and may also lessen the psychotic experiences. The recommended first line treatment for the most common sleep problem, insomnia, is cognitive‐behavioural therapy (CBT; Mitchell, Gehrman, Perlis, & Umscheid, 2012). There is evidence that for patients with psychosis, this treatment does improve sleep and may also reduce delusions and hallucinations (Myers, Startup, & Freeman, 2011). Recently, we have conducted a pilot randomized controlled trial (RCT) targeting sleep problems in 50 patients with delusions and hallucinations in the context of non‐affective psychosis (Freeman *et al*., 2013). Uptake of CBT for sleep problems was very high. The outcome data indicated that CBT, with adaptations for this population, is likely to have large benefits for improving sleep (Cohen's *d* effect size = 1.9, 95% CI = 0.9, 2.9; Freeman *et al*., in press).

Despite developments in identifying the prevalence of sleep disturbance amongst patients with psychosis, there is a lack of research exploring the patient perspective. This is especially important as accounts from non‐psychosis populations highlight that insomnia has a major impact on quality of life, but is often not understood and is assumed to be a secondary consequence of other mental and physical health conditions including depression (Carey, Moul, Pilkonis, Germain, & Buysse, 2005) and cancer (Fleming, Gillespie, & Espie, 2010). In a narrative review of patient perspectives on sleep problems and their treatment, themes of daytime impairment and difficulties accessing health care were highlighted (Cheung, Bartlett, Armour, & Saini, 2013). However, only one of the 43 studies included in the review involved participants who experienced sleep disturbance in the context of other mental health problems (Collier, Skitt, & Cutts, 2003). Therefore, the experience of sleep disturbance and its treatment in patients with psychosis remains to be explored.

In this emerging area, patients' descriptions of their experiences of sleep problems, potential interactions with psychotic experiences, and experience of evidence‐based treatment for sleep have yet to be reported. They are likely to prove an important guide for developments in understanding and treating sleep problems in psychosis. Therefore, the aim of this study was to further understand the experience of sleep problems in psychosis and the potential therapeutic benefit and experience of CBT for people with psychosis. This study uses interpretative phenomenological analysis (IPA) to explore first person accounts of sleep disturbance and therapy targeted at improving sleep in the context of current psychosis.

## Method

### Participants

Ten patients with psychosis who had recently received cognitive‐behavioural treatment for insomnia took part. The participants were recruited from the active arm of a RCT testing CBT for insomnia for patients with delusions and/or hallucinations (Freeman *et al*., 2013, in press). See Table 1 for clinical details.

**Table 1:** Clinical details of the participants

Participant number	Age (years)	Gender	Ethnicity	Clinical team diagnosis	Number of treatment sessions	Baseline assessment			Post‐treatment
						Hallucinations present (≥4 PANSS)	Persecutory delusions present (≥4 PANSS)	Insomnia Severity Index score	Insomnia Severity Index score
1	34	Female	White	Schizophrenia	7	Yes	Yes	11	6
2	19	Male	White	First Episode Psychosis	7	Yes	Yes	16	13
3	34	Female	White	Schizophrenia	9	Yes	Yes	19	1
4	46	Female	Chinese	Schizoaffective disorder	11	Yes	Yes	20	7
5	33	Male	Black	Schizophrenia	10	Yes	Yes	27	0
6	43	Female	White	Schizophrenia	9	Yes	Yes	14	8
7	65	Female	White	Schizophrenia	8	Yes	Yes	20	9
8	51	Male	White	Schizophrenia	7	Yes	Yes	20	9
9	43	Female	White	Schizoaffective disorder	8	Yes	Yes	17	11
10	20	Female	White	First Episode Psychosis	8	Yes	Yes	20	3

Note

PANSS, Positive and Negative Symptoms Scale (Kay, 1991).

### Intervention

The aim was to provide the insomnia intervention in eight sessions over twelve weeks, allowing for individual flexibility in the exact number of sessions. Patients were seen individually. The intervention is written in a manual. The main techniques, which are standard sleep interventions, were taken from four main sources (Espie, 2006; Freeman & Freeman, 2010; Harvey, Sharpley, Ree, Stinson, & Clark, 2007; Meir & Kryger, 2004). Initially, the sessions focused upon psycho‐education about sleep difficulties, assessment of the triggering and maintenance of sleep difficulties, and goal setting. Sleep diaries were kept, and some patients used actigraphy watches during therapy. Key therapeutic techniques included the following: Stimulus control (setting appropriate and regular sleep times, not doing anything else in the bed or bedroom apart from sleeping, not staying in bed if not able to sleep for longer than 20–30 min, stopping daytime naps); stabilizing circadian rhythms (e.g., gradually resetting sleep and wake times for those patients with phase disorders, exposure to sunlight in the morning, regular mealtimes); sleep hygiene and relaxation; increasing daytime activity; and, less often, cognitive techniques to address unhelpful beliefs about sleep, attentional bias, and safety behaviours. Key adaptations to the treatment have been identified (Waite *et al*., 2016).

### Procedure

Ethical approval for this study was obtained from an NHS research ethics committee. The interviews were conducted at participants' homes or at the local clinical team and ranged between 25 and 69 min. The interviews were conducted and audio‐recorded by N. Evans, an independent research worker, and transcribed verbatim.

### Semi‐structured interview

The qualitative method of IPA focuses on understanding the lived experience and how an individual makes sense of that experience (Smith, 1996). A semi‐structured interview was developed by D. Freeman, N. Evans, H. Startup, E. Myers, using guidance on IPA methodology (Smith, Flowers, & Larkin, 2009). This included open‐ended questions on three main areas: The nature of the sleep problem (e.g., ‘What was your sleep like before the therapy?'); any changes following therapy (e.g., ‘After having had the therapy, what is your sleep like now?'); and the experience of taking part in therapy (e.g., ‘How was your experience of this therapy?'). The interview schedule was used flexibly, additional verbal and non‐verbal cues were given to encourage elaboration, and participants' own vocabulary was used. In addition, participants completed a 4‐point Likert scale indicating their overall satisfaction with therapy.

### Analysis

Interpretative phenomenological analysis is an idiographic approach focused on how an individual experiences and makes sense of a specific phenomenon, and is considered a helpful method to explore the experience and treatment of psychosis (Kilbride *et al*., 2013; Smith *et al*., 2009) and the experience and treatment of sleep problems (Carey *et al*., 2005; Kyle, Morgan, & Espie, 2010; Kyle, Morgan, Spiegelhalder, & Espie, 2011). Analysis followed the procedure outlined by Smith *et al*. (2009) and was primarily conducted by F. Waite and N. Evans. F. Waite and N. Evans completed independent analysis on each transcript before discussions to consider credibility of the coding. F. Waite completed a reflexive log throughout analysis. Initial exploratory comments were noted and these were developed into emergent transcript themes. Through an iterative process, the emergent themes were clustered and organized into superordinate themes. The superordinate themes were reviewed by D. Freeman. Discrepancies between interpretations were discussed and the transcripts revisited. Similarities and differences in accounts, both within and between participants, were explored to ensure the themes were grounded in the data. The cross checking of data by different researchers within the group provided opportunities to enhance the credibility of the findings. The Yardley (2000, 2008) criteria for enhancing quality in qualitative research were used throughout the study. The criteria include sensitivity to context, commitment and rigour, transparency and coherence, and impact and importance. Specific adaptations of these criteria for IPA were informed by Smith *et al*. (2009).

## Results

Six superordinate themes were identified (Table 2). The first three superordinate themes concerned the experience of sleep problems and their interaction with psychosis, and the last three themes concerned the treatment experience. The superordinate and related subordinate themes (noted in the main text in italics) are presented by research question and discussed in detail below. To ensure transparency, anonymized quotes (with reference to the participant number) are used to reflect participants' experiences.

**Table 2:** Overview of superordinate and subordinate themes

Superordinate and subordinate themes
Nature of sleep problems: ‘Trying to sleep but I couldn't' (5) Impact of sleep problems: ‘When I'm tired, everything is worse' (1) Interaction of sleep problems and psychosis: ‘A nightmare you can't get out of' (4) Positive changes following therapy: ‘It sorted out my sleep and I am a better person for it' (10) Developing new knowledge and skills: ‘Tools for my sleep' (9) Easy and understandable process: ‘Straightforward really, really easy to learn' (1) Effortful and challenging process: ‘I stuck with it' (4) Therapy style: ‘A different approach' (10)

Note

Participant number reported for each quotation.

### Experience of sleep problems

#### The nature of sleep problems: ‘Trying to sleep but I couldn't' (Participant 5)

The first superordinate theme captured descriptions of the nature of sleep problems. Participants' described experiences of insomnia: ‘I used to stay in bed trying to get some sleep for hours, things going through my mind. I used to wake up in the middle of the night and I used to get up very early in the morning as well' (8).

A feature of sleep disturbance was the frustration and effort to attempt to sleep: ‘It was frustrating because I was trying to sleep and couldn't sleep' (5), ‘it would bother me that I hadn't gone to sleep and I would get more worked up about it, which means I was less likely to get to sleep' (2). Worry was described as a major cause of disruption to sleep, ‘I used to sit and worry all night. I used to use that as my worry time' (10). The content of worry included daily tasks, finances, and living situation, although for some participants the worries focused specifically on fears of persecution from others, ‘I always think people are trying to kill me' (8) or concerns about relapse and psychotic symptoms, ‘suddenly start worrying that I didn't have enough medication' (4).

Sleep was described as broken and often featured distressing dreams and nightmares: ‘I got broken sleep; I kept waking up and going in to dreams' (7). Nightmares further disrupted sleep by causing sleep avoidance: ‘Bad nightmares and night terrors and so I tried to stay awake longer' (2).

Participants reported circadian rhythm disruption (changes to their body clocks), describing their sleep as ‘very erratic' (6), ‘all over the place' (3), ‘it was so hectic, it was so messed up' (4). Further disruption to circadian rhythm was caused by daytime naps used to manage poor sleep: ‘Because I was getting less sleep it made me take more naps in the day' (5). Participants reported a phase advance, going to bed very early, or phase delay, going to bed very late, or an erratic pattern with a mixture of too little and too much sleep. These extended periods of sleep or hypersomnia disrupted the daytime schedule, for example having ‘breakfast at 2 pm' (3).

#### Impact of sleep problems: ‘When I'm tired, everything is worse' (1)

The second superordinate theme concerned the impact of sleep problems on the patients' lives. This included emotional distress, fatigue, and lost opportunities for daytime activities. Participants noted a range of emotional responses to sleep problems, including increased agitation, ‘bit more agitated if I haven't slept for in 2 or 3 days' (2), ‘I was stroppy all the time' (10) and increased anxiety, ‘I am more nervous and worried if I don't get sleep' (7). The pervasive fatigue, ‘I was shattered all the time' (1), was so disruptive one participant commented, ‘I am going to fall asleep here' (9) during the interview.

One of the most striking features of participants' accounts was the impact on daytime functioning. Participants described ‘missing out' (1) on daytime activities, ‘it annoyed me, because I was just wasting time really being asleep all day' (3). Sleep disturbance reduced participants' opportunities to complete daily tasks: ‘It wasn't giving me enough time to do what I wanted during the day' (3), ‘I used to do my shop during the night because I didn't have time to do it during the day' (10). Sleep disturbance had an impact on social relationships, ‘it was affecting my social life' (10). In contrast, however, two participants described ‘leading life normally' (2) despite sleep disturbance. These participants noted, ‘it wasn't really bothering me because I got used to it' (2) and therefore ‘I was still able to do things during the day' (6).

#### Interaction of sleep problems and psychosis: ‘A nightmare you can't get out of' (4)

Participants described a bidirectional causal relationship between sleep disturbance and psychotic experiences. Participants described how, ‘the voices wrecked my sleep' (4) in particular distressing voices prevented sleep onset: ‘Trying to sleep and they [the voices] distract me and they keep me awake' (5). The distressing nature of hearing voices led to frustration, hyperarousal and delayed sleep onset: ‘Annoying, frustrating, makes you want to pull your hair out, especially when you're trying to sleep at night and he [the voice] won't shut up talking… It is hard to try and go to sleep if he is in one of his annoying moods because my mind can't switch off because although I am not trying to concentrate on him, when someone speaks you automatically hear what they are saying. So it is like that for me, I still register what he says and then my mind is still thinking and then I think about what he says. It is quite difficult in that respect and that hasn't helped my sleep' (10). These experiences led to fear and anxiety which further disrupted sleep, ‘he [the voice] makes weird shadows when I am trying to sleep and it kind of freaks me out' (10). Beliefs about persecution resulted in further disruption to sleep: ‘They [the voices] would make me lie down between 4 pm and 5 pm or else they said they would kill me if I didn't' (7). Avoidance of sleep resulted from fears related to plots or schemes developed, while the participant was asleep, for example ‘they have had time to get it together' (3).

Sleep problems reduced participants' capacity to cope with distressing voices: ‘When I'm tired it gets worse because I don't have the strength to fight them as much' (1) and strengthened conviction in fears of persecution, ‘the more tired I am the worse they get' (3). Sleep was identified as a way of improving psychotic experiences, ‘sometimes my voices would be better as well from sleeping' (2). Thus, participants' accounts sometimes noted a bidirectional relationship between sleep problems and psychotic experiences.

Sleep was identified as a way of coping but also as an escape from psychotic experiences. Participant 7 spoke extensively about finding respite from psychotic experiences in bed, ‘I was alright once I got in to bed…they [the voices] would usually go on, but it doesn't affect me in bed. It is about the only place it doesn't' (7). The participant described beliefs about why sleep provided a respite from psychotic experiences, ‘they [the voices] fly out of your head when you are asleep' (7). This reflects the intricate relationship between sleep disturbance and psychotic experiences.

### Experience of therapy

#### Positive changes following therapy: ‘It sorted out my sleep and I am a better person for it' (10)

The fourth superordinate theme concerned the breadth of positive changes following the therapy. Many participants reported improvements in their sleep, their capacity to cope with psychotic experiences, their general well‐being, including mood, hope for the future, and sense of self and finally improvements in social functioning.

Participants' reported clear and meaningful improvements in sleep: ‘I am getting better sleep' (3). This included the following: ‘I go to sleep earlier' (7), ‘I tend to sleep for longer and it is not so broken' (7), and ‘I don't feel so tired now' (7). These changes were noted as significant improvements, ‘it is completely different now' (10). There was a shift from the effortful, frustrated attempts to sleep, to a natural occurrence of sleep, ‘I just sleep' (9), ‘I don't get as worked up about it and I do get to sleep more often now' (2). Bed was re‐associated with sleep, ‘when I am tired I go to bed and I can sleep' (6), and there was a reduction in night time worry, ‘I can switch my mind off' (10). In summary, participants stated that the intervention improved sleep: ‘It has helped me to sleep' (6).

Improvements in psychotic experiences were reported by a number of participants, and these changes were specifically attributed to improved sleep: ‘Calming my voices in my mind down as well because I am sleeping a lot more' (5). Other participants noted that improved sleep led to increased resources to cope with and challenge psychotic experiences: ‘The way I try and manage and cope with him [the voice] is different' (10). However, some participants were cautious about not overselling the improvements, ‘it's not as bad as it was' (1) and noted that they continued to struggle with managing psychotic experiences for example fears of persecution restricting daytime activity, ‘I still find that quite difficult' (3). Participants' accounts highlighted improvements in psychotic experiences, reduction in frequency and distress, increased capacity for and ways of coping ‘If I sleep I can do everything better' (10), ‘I feel more in control, I feel I can do things' (3); however, no participants reported a complete absence of distressing psychotic experiences after treatment.

The improvements in sleep led to wider improvements, ‘I feel a bit better now I am sleeping better' (2). These included improvements in well‐being, ‘I feel stronger mentally and physically' (7), reduced anxiety ‘I am less anxious, a lot calmer' (5) improved mood and hope for the future, ‘I wake up feeling more optimistic and happier' (2), a sense of normality or being normal, ‘I feel a bit more normal' (3), ‘live a normal life again and am being a normal person' (4) and improved self‐esteem, ‘I wake up now feeling a lot better about myself' (2).

Daily functioning changed following therapy, ‘my whole lifestyle has changed' (10). This increase in activity, for example ‘my days are busier' (1), included practical adjustments, ‘more time in the morning to get things done' (7), social changes, ‘doing a lot more with my friends' (3) and increases in meaningful activity, ‘signed up to go back to college' (2). In sum, participants noted that sleep was a key factor for a range of significant outcomes: ‘Teaching me how to sleep, how to be well' (4).

Participants made clear attributions of these wider positive outcomes to improvements in sleep: ‘Because of sleep, because then you have more energy to tackle the problem, I think it is the sleep that helped me this time' (4), ‘it must be my sleep' (1). However, others noted that the changes were ‘intertwined' (5) as, ‘sorting out one thing meant I could address another' (5).

#### Developing new knowledge and skills: ‘Tools for my sleep' (9)

The fifth superordinate theme concerned the process of developing new skills for improving sleep. It features two contrasting elements: *5a Easy and understandable process:* ‘Straightforward really, really easy to learn' *(1)* and *5b Effortful and challenging process*: ‘I stuck with it' *(4)*.

Participants often described developing a new understanding of their sleep problems based on the sleep diaries, ‘from the sleep pattern you can see your mind and body reaction in the chart' (4). Reviewing material related to causes of sleep disturbance aided development of a clear formulation and rationale for treatment, for example ‘we went a lot through the routes and causes of my sleep problems to try and solve those' (2) and ‘my pattern had shifted like jet lag, and that was why I couldn't sleep at night and then was waking up late in the day. So, we would move it gradually back and that really worked really well' (3). This process ‘was interesting' (8) and participants noted the importance of personal understanding as well as clarity of ideas, ‘it all makes sense, and it all made sense to me' (3).

Participants described the volume of strategies developed in therapy: ‘There were absolutely loads of techniques' (10). There was a split between those participants describing the range of strategies as important, ‘all the tools, all of it' (3) and those who identified one key strategy, for example, ‘cutting out the coke [drink]' (6) or ‘condensing the sleep' (7). For these participants, it was not the breadth or range of strategies but rather the application of one specific strategy which led to meaningful improvements in their sleep.

The varied accounts of key therapy strategies reflected the personalized formulations of maintenance factors. The key strategies included ‘getting in to a routine, the rise up and bedtime routine' (9), ‘the routine that is what really made the difference' (8), reducing worry and anxiety including using relaxation exercises/CD, ‘that helped to calm me down' (5) and increasing daytime activity, ‘went for walks and that, it tires me out' (9). However, the main strategy dominating participants' accounts was the use of stimulus control and their newly developed ‘sleeping zone' (10): ‘Just associating the bedroom with sleep rather than watching TV' (8).

Participants noted that the recovery trajectory was not simple, ‘to start with it didn't go well, it went worse, then it just got better and better' (4) and thus therapy required effortful determination to overcome challenges. Goal setting followed by monitoring progress helped to boost motivation, ‘I enjoyed trying to achieve my goals and achieving them' (10). This included using actigraphy for feedback, ‘using the [actigraphy] watch data as well to actually see the changes to see how much you are improving, that helped because it does fill you with a bit of confidence when you can see physically see that it is improving' (2). Participants described a sense of personal responsibility and effort for change, ‘I tried hard at this stuff' (5).

Some participants described challenges in the therapy process. This included challenges to motivation ‘quite hard to keep myself motivated to do it' (2), difficulties remembering to implement strategies ‘I keep forgetting to do them' (7) or not attempting strategies, ‘I haven't tried it yet' (9). However, participants also described how psychotic experiences influenced therapy, ‘[the voice] sometimes doesn't like the fact that I am trying to sort out my sleep and sort out other things and he is quite nasty about it sometimes' (10). For some participants, this led to adaptations in therapy, for example, ‘the therapist said you should get up and do something instead of just lying there and I said that the voices wouldn't let me. So the therapist said you have to do the relaxation exercises in bed instead' (7). This reflects the adaptations which may be necessary and the importance of flexibility to overcome the influence of psychotic experiences on the treatment.

#### Therapy style: ‘A different approach' (10)

The sixth superordinate theme concerned the style of the intervention. The therapy experience was described in contrast to participants' previous experiences of help. Previous input often focused on the use of medication or sleep hygiene reading materials which was not perceived as particularly beneficial, ‘did help a little bit but it didn't make a lot of difference' (2). The previous experiences of help influenced participants' treatment expectations: ‘I didn't think it would work, because nothing else had worked so far' (1). In contrast, ‘I had CBT before and that worked for some other things that I had problems with so I thought it would be a good idea to give it a go' (8). Some participants were hopeful about treatment, ‘I believed it would help me with my sleep' (6). However, others were willing to engage but did not expect changes: ‘I will give it a shot, but it is not going to work' (10). The willingness to engage with the intervention was driven by the combination of a desire for help, frustration and a lack of other options, for example participants said, ‘I've got to try something' (8) and ‘I've got nothing to lose' (1). Indeed participants noted it was ‘a refreshing changes to get some help' (7).

This intervention was seen as distinct from previous experiences of help as it was ‘a different approach' (10). Participants described an overall positive experience of the therapy, ‘it was really, really good. Really good. I was really impressed. I didn't think it was going to help or anything. I am really impressed' (10). The therapy was perceived as client led, ‘on your own terms instead of feeling you have to do it' (10), and active, ‘a “try and do” therapy' (10). This practical and active approach, for example going for walks, was valued by participants. One participant noted the sessions which were less helpful were when there was, ‘too much talking!' (9).

The therapeutic alliance was highlighted by all participants. Specific skills contributed to the development of a strong therapeutic alliance, for example active listening: ‘She listened; she took the things I said on board' (2). Participants described a sense of connection and being cared for by the therapist, ‘she was really easy to get on with, really easy to talk to and she actually really did care and she really actually did want to help, which I had never really got that sort of vibe off anyone else before. I think she made the therapy really good. She stood out and really made everything else stand out' (10). This example highlights how the therapeutic alliance and attributes of the therapist influenced the treatment experience and efficacy.

The commitment of the therapist facilitated engagement and motivation, for example, ‘she wanted to help me, she was efficient, enthusiastic' (6). Collaboration was facilitated by developing shared rationales and understanding: ‘She was very open and honest with me about what she was going to do, or try to do' (1). Participants described collaboration as joint working, for example: ‘We worked it out' (3). Collaboration also included the therapist acting as a support, ‘she was always there to back me up' (1). This interaction between therapist and participant resulted in empowerment, ‘because of her I helped myself' (4).

The strong therapeutic alliance and active focus were highly valued by participants. The overall experience of this new approach was described as very helpful. One participant commented, ‘I'd happily do it a million times over' (10).

### Satisfaction with treatment

Participants rated their satisfaction/dissatisfaction with the treatment on a 4‐point Likert scale. A total of eight participants rated being ‘very satisfied' (score of 4) and two participants rated being ‘satisfied' (score of 3) with the treatment.

## Discussion

This is the first report of patient experiences of sleep problems in the context of psychosis. Ten patients who had delusions and hallucinations, sleep problems, and received psychological help for insomnia were interviewed. The accounts were strikingly rich, providing strong indications of the variety of sleep problems experienced in psychosis, the significant impact that they have on day‐to‐day life, and the positivity from patients about receiving an optimal intervention for their concerns. A bidirectional link between sleep and psychotic experiences was reported, supporting both models specific to psychosis (Freeman *et al*., 2010) and the wider view that sleep disturbance and mental health interact reciprocally via changes in mood regulation, cognitive functioning, and daily activity (Harvey, 2008).

The negative daytime effects or impact of sleep problems identified are similar to those reported in previous IPA research exploring the experiences of people with persistent insomnia (Carey *et al*., 2005; Kyle *et al*., 2010). The key areas of similarity relate to those aspects of the daytime effects which adjusted following therapy, for example increased social contact, feeling more energized and engaging with activities, such as returning to college. In contrast, the impact and interaction of psychosis on sleep are novel features which have not been previously reported in qualitative research.

With regard to the experience of treatment, there are similarities with previous IPA research in the field of cognitive‐behavioural therapy for psychosis (CBTp; Kilbride *et al*., 2013) and CBT for insomnia (Kyle *et al*., 2011). In their user‐led IPA paper exploring CBTp, Kilbride *et al*. (2013) detail the importance of a clear, understandable, and engaging approach. Similarities with themes identified from an IPA study of the experience of sleep restriction therapy (Kyle *et al*., 2011) include the negative effects and desperation for help (‘at the end of my tether'), challenges to engaging with strategies (‘adherence and adjustment'), positive changes to sleep (‘actually want to go to bed now'), and wider outcomes including feeling calmer, enhanced motivation, and increased daily activity (‘daytime functioning: A thermometer for success?'). This suggests similarities in the experience of therapies for sleep problems amongst people with and without experiences of psychosis and that an engaging and understandable approach is valued when working with people with psychosis. However, specific consideration of how psychotic experiences impact on sleep and its treatment are unique features which have not been previously reported and indicate a potentially important area for future research.

The participants are a selected group, in the context of a research trial, and therefore, there is clearly a potential bias in their experiences. However, IPA research does not aim to produce generalizable results but rather inform theoretical and clinical applications from first person accounts. We consider that the accounts described in this paper will prove a valuable resource that will meet such aims. Where the bias is most likely to occur is the enthusiasm for the psychological treatment. The positive experiences of treatment described by the participants in this study may not reflect the wider population. However, there is a substantial literature showing the benefits of such interventions for insomnia (Sivertsen *et al*., 2006). Our work gives every indication that it is applicable, suitably modified, to patients with psychosis (Freeman *et al*., in press; Myers *et al*., 2011). There was clear appreciation by these patients for practical, collaborative treatment work on an important problem to them.

This is the first published account of patients' experiences of sleep problems and their treatment in people with psychosis. The themes identified indicate that sleep disturbance is a major problem which interacts in a bidirectional relationship with psychosis. Treatment is desired and previous help has either been unsuccessful or lacking. The current CBT intervention was valued by participants and led to a range of reported positive outcomes. Our view is that sleep problems in psychiatric disorders need much greater emphasis in research and treatment.

## Funding

The work is funded by a grant from the NHS NIHR Research for Patient Benefit Programme (reference PB‐PG‐0211‐10007). DF is supported by a Medical Research Council Senior Clinical Fellowship (G0902308). Research support to DF, FW, and RL is also provided by a Wellcome Trust Strategic Award (098461/Z/12/Z) for the Oxford Sleep and Circadian Neurosciences Institute.

## Author contributions

DF, NE, HS, and EM developed the interview for data collection. RL and NE recruited participants for the study. EM and FW provided the intervention. DF, HS, and AH provided clinical supervision. NE conducted the interviews for data collection. FW and NE conducted the analysis. DF provided overall supervision. FW and DF took the main responsibility for drafting of the manuscript. All authors read and approved the final manuscript.

## References

[papt12073-bib-0001] Anderson, K. N. , & Bradley, A. J. (2013). Sleep disturbance in mental health problems and neurodegenerative disease. Nature and Science of Sleep, 5, 61–75. doi:10.2147/NSS.S34842 10.2147/NSS.S34842 PMC3674021 23761983

[papt12073-bib-0002] Baglioni, C. , Battagliese, G. , Feige, B. , Spiegelhalder, K. , Nissen, C. , Voderholzer, U. , … Riemann, D. (2011). Insomnia as a predictor of depression: A meta‐analytic evaluation of longitudinal epidemiological studies. Journal of Affective Disorders, 135(1–3), 10–19. doi:10.1016/j.jad.2011.01.011 21300408 10.1016/j.jad.2011.01.011

[papt12073-bib-0003] Carey, T. J. , Moul, D. E. , Pilkonis, P. , Germain, A. , & Buysse, D. J. (2005). Focusing on the experience of insomnia. Behavioral Sleep Medicine, 3(2), 73–86. doi:10.1207/s15402010bsm0302_2 15802258 10.1207/s15402010bsm0302_2

[papt12073-bib-0004] Cheung, J. M. Y. , Bartlett, D. J. , Armour, C. L. , & Saini, B. (2013). The insomnia patient perspective, a narrative review. Behavioral Sleep Medicine, 11, 369–389. doi:10.1080/15402002.2012.694382 23402684 10.1080/15402002.2012.694382

[papt12073-bib-0005] Collier, E. , Skitt, G. , & Cutts, H. (2003). A study on the experience of insomnia in a psychiatric inpatient population. Journal of Psychiatric and Mental Health Nursing, 10, 697–704. doi:10.1046/j.1365‐2850.2003.00654.x 15005483 10.1046/j.1365-2850.2003.00654.x

[papt12073-bib-0006] Espie, C. A. (2006). Overcoming insomnia and sleep problems: A self help guide using cognitive behavioural techniques. London, UK: Constable and Robinson.

[papt12073-bib-0007] Fleming, L. , Gillespie, S. , & Espie, C. A. (2010). The development and impact of insomnia on cancer survivors: A qualitative analysis. Psycho‐Oncology, 19, 991–996. doi:10.1002/pon.1652 20014075 10.1002/pon.1652

[papt12073-bib-0008] Freeman, D. , Brugha, T. , Meltzer, H. , Jenkins, R. , Stahl, D. , & Bebbington, P. (2010). Persecutory ideation and insomnia: Findings from the second British National Survey Of Psychiatric Morbidity. Journal of Psychiatric Research, 44, 1021–1026. doi:10.1016/j.jpsychires.2010.03.018 20434169 10.1016/j.jpsychires.2010.03.018 PMC2977847

[papt12073-bib-0009] Freeman, D. , & Freeman, J. (2010). Know your mind: The complete family reference guide to emotional health. New York, NY: Sterling.

[papt12073-bib-0010] Freeman, D. , Pugh, K. , Vorontsova, N. , & Southgate, L. (2009). Insomnia and paranoia. Schizophrenia Research, 108(1–3), 280–284. doi:10.1016/j.schres.2008.12.001 19097752 10.1016/j.schres.2008.12.001 PMC2697325

[papt12073-bib-0011] Freeman, D. , Stahl, D. , McManus, S. , Meltzer, H. , Brugha, T. , Wiles, N. , & Bebbington, P. (2012). Insomnia, worry, anxiety and depression as predictors of the occurrence and persistence of paranoid thinking. Social Psychiatry and Psychiatric Epidemiology, 47, 1195–1203. doi:10.1007/s00127‐011‐0433‐1 21928153 10.1007/s00127-011-0433-1

[papt12073-bib-0012] Freeman, D. , Startup, H. , Myers, E. , Harvey, A. , Geddes, J. , Yu, L.‐M. , … Lister, R. (2013). The effects of using cognitive behavioural therapy to improve sleep for patients with delusions and hallucinations (the BEST study): Study protocol for a randomized controlled trial. Trials, 14(1), 214. doi:10.1186/1745‐6215‐14‐214 23845104 10.1186/1745-6215-14-214 PMC3717119

[papt12073-bib-0013] Freeman, D. , Waite, F. , Startup, H. , Myers, E. , Lister, R. , McInerney, J. , … Yu, L.‐M. (in press). A randomi sed controlled pilot trial testing the effects of cognitive behavioural therapy to improve sleep for patients with persistent delusions and hallucinations: The Better Sleep Trial (BEST). The Lancet Psychiatry. 10.1016/S2215-0366(15)00314-4 PMC4641164 26363701

[papt12073-bib-0014] Harvey, A. G. (2008). Insomnia, psychiatric disorders, and the transdiagnostic perspective. Current Directions in Psychological Science, 17, 299–303. doi:10.1111/j.1467‐8721.2008.00594.x

[papt12073-bib-0015] Harvey, A. G. , Sharpley, A. L. , Ree, M. J. , Stinson, K. , & Clark, D. M. (2007). An open trial of cognitive therapy for chronic insomnia. Behaviour Research and Therapy, 45, 2491–2501. doi:10.1016/j.brat.2007.04.007 17583673 10.1016/j.brat.2007.04.007

[papt12073-bib-0016] Hofstetter, J. R. , Lysaker, P. H. , & Mayeda, A. R. (2005). Quality of sleep in patients with schizophrenia is associated with quality of life and coping. BMC Psychiatry, 5, 13. doi:10.1186/1471‐244X‐5‐13 15743538 10.1186/1471-244X-5-13 PMC554780

[papt12073-bib-0100] Kay, S. R. (1991). Positive and negative syndromes in schizophrenia. New York, NY: Brunner.

[papt12073-bib-0017] Kilbride, M. , Byrne, R. , Price, J. , Wood, L. , Barratt, S. , Welford, M. , & Morrison, A. P. (2013). Exploring service users' perceptions of cognitive behavioural therapy for psychosis: A user led study. Behavioural and Cognitive Psychotherapy, 41, 89–102. doi:10.1017/S1352465812000495 22874770 10.1017/S1352465812000495

[papt12073-bib-0018] Kyle, S. D. , Morgan, K. , & Espie, C. A. (2010). Insomnia and health‐related quality of life. Sleep Medicine Reviews, 14(1), 69–82. doi:10.1016/j.smrv.2009.07.004 19962922 10.1016/j.smrv.2009.07.004

[papt12073-bib-0019] Kyle, S. D. , Morgan, K. , Spiegelhalder, K. , & Espie, C. A. (2011). No pain, no gain: An exploratory within‐subjects mixed‐methods evaluation of the patient experience of sleep restriction therapy (SRT) for insomnia. Sleep Medicine, 12, 735–747. doi:10.1016/j.sleep.2011.03.016 21907616 10.1016/j.sleep.2011.03.016

[papt12073-bib-0020] Meir, H. , & Kryger, M. (2004). A woman's guide to sleep disorders. New York, NY: McGraw‐Hill.

[papt12073-bib-0021] Mitchell, M. D. , Gehrman, P. , Perlis, M. , & Umscheid, C. A. (2012). Comparative effectiveness of cognitive behavioral therapy for insomnia: A systematic review. BMC Family Practice, 13(1), 40. doi:10.1186/1471‐2296‐13‐40 22631616 10.1186/1471-2296-13-40 PMC3481424

[papt12073-bib-0022] Monti, J. M. , & Monti, D. (2005). Sleep disturbance in schizophrenia. International Review of Psychiatry (Abingdon, England), 17, 247–253. doi:10.1080/09540260500104516 10.1080/09540260500104516 16194796

[papt12073-bib-0023] Myers, E. , Startup, H. , & Freeman, D. (2011). Cognitive behavioural treatment of insomnia in individuals with persistent persecutory delusions: A pilot trial. Journal of Behavior Therapy and Experimental Psychiatry, 42, 330–336. doi:10.1016/j.jbtep.2011.02.004 21367359 10.1016/j.jbtep.2011.02.004 PMC3566479

[papt12073-bib-0024] Ruhrmann, S. , Schultze‐Lutter, F. , Salokangas, R. K. R. , Heinimaa, M. , Linszen, D. , Dingemans, P. , … Klosterkötter, J. (2010). Prediction of psychosis in adolescents and young adults at high risk: Results from the prospective European prediction of psychosis study. Archives of General Psychiatry, 67, 241–251. doi:10.1001/archgenpsychiatry.2009.206 20194824 10.1001/archgenpsychiatry.2009.206

[papt12073-bib-0025] Sivertsen, B. , Omvik, S. , Pallesen, S. , Bjorvatn, B. , Havik, O. , Kvale, G. , … Nordhus, I. (2006). Cognitive behavioral therapy vs zopiclone for treatment of chronic primary insomnia. Journal of the American Medical Association, 295, 2851–2858. doi:10.1001/jama.295.24.2851 16804151 10.1001/jama.295.24.2851

[papt12073-bib-0026] Smith, J. A. (1996). Beyond the divide between cognition and discourse: Using interpretative phenomenological analysis in health psychology. Psychology & Health, 11, 261–271. doi:10.1080/08870449608400256

[papt12073-bib-0027] Smith, J. A. , Flowers, P. , & Larkin, M. (2009). Interpretative phenomenological analysis: Theory, method and research. London, UK: Sage.

[papt12073-bib-0028] Waite, F. , Myers, E. , Harvey, A. G. , Espie, C. A. , Startup, H. , Sheaves, B. , & Freeman, D. (2016). Treating sleep problems in patients with schizophrenia. Behavioural and Cognitive Psychotherapy, 44, 273–287. doi:10.1017/S1352465815000430 26751571 10.1017/S1352465815000430 PMC4855992

[papt12073-bib-0029] Wulff, K. , Dijk, D.‐J. , Middleton, B. , Foster, R. G. , & Joyce, E. M. (2012). Sleep and circadian rhythm disruption in schizophrenia. The British Journal of Psychiatry, 200, 308–316. doi:10.1192/bjp.bp.111.096321 22194182 10.1192/bjp.bp.111.096321 PMC3317037

[papt12073-bib-0030] Yardley, L. (2000). Dilemmas in qualitative health research. Psychology & Health, 15, 215–228. doi:10.1080/08870440008400302

[papt12073-bib-0031] Yardley, L. (2008). Demonstrating validity in qualitative health research In Smith J. A. (Ed.), Qualitative psychology: A practical guide to methods (2nd ed., pp. 235–251). London, UK: Sage.

